# Supplementary material for: Complexity and Community Context: Learning from the Evaluation Design of a National Community Empowerment Programme
Source: Int J Environ Res Public Health. 2019 Dec 21;17(1):91. doi: 10.3390/ijerph17010091 (PMC6981559; doi:10.3390/ijerph17010091)
Supplement: Supplementary file 1 [file ijerph-17-00091-s001.pdf]

Table S1 Research questions linked to Local People theory of change

Research Questions	Key areas of measurement (literature review)	Local People Theory of change
How does the LP approach work at a local level as a way of developing collective control? What are the barriers and facilitators?	Contextual factors<br>Community assets<br>Capacity building/engagement (how)<br>Barriers and facilitators	Inputs<br>• National organisations<br>• Grant money<br>• Local assets
What are the pathways of engagement and change within neighbourhoods and communities of interest? What contextual factors influence these pathways?	Contextual factors<br>Community engagement & social action<br>Inequalities and reach<br>Ladder of engagement	Mechanisms of change<br>• Engagement & outreach<br>• Coming together<br>• Dialogue and decision making
Do residents achieve the shorter term outcomes outlined in the ToC? Are there unintended outcomes?	Individual empowerment/confidence<br>Personal and collective resources<br>Social networks & social capital- bonding and bridging; sense of belonging and trust; reduction of social isolation<br>Civic participation<br>Better access to services	Shorter term changes<br>• Social connectedness<br>• Knowledge, understanding and skills<br>• Confidence and aspiration<br>• Involvement and influence
How do residents view the experience of and outcomes from participation (or non-participation) in Local People within the context of community life?	Experience of power & powerlessness<br>Participation – who, why and how?	Individual action<br>Collective action
What are the economic and social impacts at a neighbourhood level resulting from participation in Local People?	Improvements in community conditions<br>Improvements in social determinants; including housing, employment, education, environment.<br>Quality of life<br>Changes to local services<br>Transformation of place/neighbourhood<br>Increased collective control	Longer term changes<br>• Better health & wellbeing<br>• Improvements in social determinants of health<br>• Better place to live<br>• Better quality of life<br>• Increased control in life
What is the evidence of increased levels of control – at personal, community and societal levels?	Increased community capabilities<br>Greater power and emancipation<br>Individual and collective agency to bring about changes in community conditions and/or society	Individual and collective action<br>Longer term changes<br>• Increased control in life

What is the role of national partners in supporting implementation and capacity building at local level? Does the federated structure of the national organisations interact with the localised model of Local People to provide added value?	Capacity building<br>Partnership working	Decreasing gradient of accountability – towards community control<br>Organisations embedding collective control
How does the organisational focus of Local People partners influence the way of working with the neighbourhood or target group of residents?	Capacity building	Organisations embedding collective control
How does working with the Trust's Local People model affect and influence national partner organisations' ways of working beyond the programme?.	Capacity building<br>Sustainability<br>Partnership working	Organisations embedding collective control
What are the key characteristics for the successful development of the Local People model, identifying features of projects that support this success?	Do any existing models fit well with our findings?	Whole ToC as a pathway to change
